# The effects of *Alkanna tinctoria* Tausch on split-thickness skin graft donor site management: a randomized, blinded placebo-controlled trial

**DOI:** 10.1186/s12906-017-1741-0

**Published:** 2017-05-08

**Authors:** Aliasghar Kheiri, Shahideh Amini, Abbas Norouzi Javidan, Mohammad Mehdi Saghafi, Ghasemali Khorasani

**Affiliations:** 1 Plastic and Reconstructive Surgery Division, Faculty of Medicine, Tehran University of Medical Sciences, Tehran, Iran; 2 Clinical Pharmacy Department, Faculty of Pharmacy, Tehran University of Medical Sciences, Tehran, Iran; 3 Brain and Spinal Injury Research Center, Institute of Neuroscience, Tehran University of Medical Sciences, Tehran, Iran; 4 Pharmacology Department, Faculty of Pharmacy, Iran University of Medical Sciences, Tehran, Iran

**Keywords:** *Alkanna*, Skin graft, Wound, Healing

## Abstract

**Background:**

A prospective, randomized, placebo-controlled clinical trial was conducted to compare the healing effectiveness of *Alkanna tinctoria* (L.) Tausch (Boraginaceae) with standard dressing on wound healing at the donor site after removal of the skin graft.

**Methods:**

Enrolled patients were randomly allocated to receive topical*A. tinctoria* extract ointment (20%) or standard dressing (dressing with base ointment) daily. Wound healing was assessed using the Bates-Jenson assessment tool at the 2^nd^ and 4^th^ weeks after intervention.

**Results:**

Decreases in wound score were significantly greater in the *A. tinctoria* group compared with the placebo group (*P <*0.05). The surface areas of graft donor sites in the *A. tinctoria* group were significantly reduced as compared with the control group at day 28 of the intervention (*P <* 0.05). The proportion of patients in the *A. tinctoria* group achieving complete wound healing within 2 to 4 weeks was 50% and 96.66%, respectively, significantly higher than in patients receiving standard care: 0% and 23.3%, respectively.

**Conclusions:**

This clinical study showed that *A. tinctoria* dressing accelerates wound healing after graft harvesting.

**Trial registration:**

IRCT ID: IRCT201511165781N2.

## Background

Split-thicknesses skin graft (STSG) is a beneficial technique used to enhance wound healing. However, donor site management after skin graft harvesting is an important issue in reconstructive surgery [1]. The ideal dressing accelerates the healing process, prevents infectious complications and is easy to apply and cost effective [2, 3]. Several dressings and topical agents have been applied clinically for wound healing at the donor site, but the optimum dressing agents or uniform standard dressing for local wound care is unclear. However, no evidence has documented any superiority or advantage of a particular dressing [2, 4, 5]. The process of wound healing after skin graft harvesting depends markedly on the extent of inflammation and infection [6, 7]. *Alkanna tinctoria* (L.) Tausch (Boraginaceae) (*A. tinctoria*) is a historical plant that has been used for the treatment of macular eruptions and infectious diseases, such as measles, sore-throat, carbuncles and burns, and has also been used as a dye in the cosmetic and fabric industries. Since ancient times, roots extracted from *A. Tinctoria* have been used to treat wounds [8, 9]. The main active ingredients of *A. Tinctoria* are Alkannin and shikonin (A/S) [8]. Alkannin derivatives show strong wound healing along with anti-inflammatory and antimicrobial properties [10, 11]. Although the wound-healing effect of *A. tinctorial* was first identified in 1976, in the era of modern medicine, the use of this plant has declined [10, 12]. The purpose of this study was to evaluate the healing effectiveness of (*A. tinctoria*) Tausch dressing for STSG donor site coverage compared with standard dressing at the donor site after removal of the skin graft.

## Methods

**Fig. 1:**
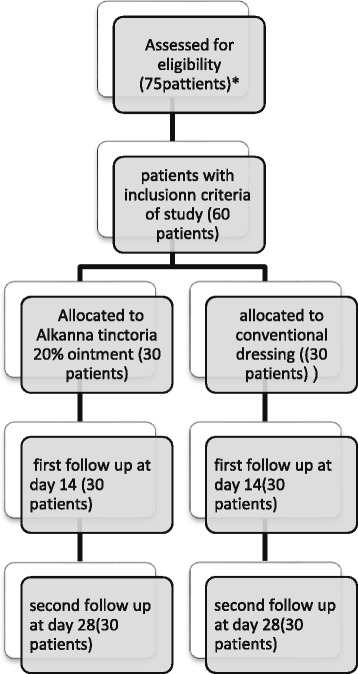
Consort flowchart of the study.*Seven diabetic patients, three patients with chronic kidney disease and five patients who were unwilling to participate in the study were excluded

A prospective, randomized, placebo-controlled clinical trial was conducted between October 2015 and January 2016 at Vali-Asr Hospital, a tertiary university hospital in Tehran. The study protocol was approved by the Ethics committee of Tehran University of Medical Science and written informed consent was obtained from all participants after registration of the study in the Iranian Registry of Clinical Trials (IRCT) (IRCT201511165781N2). Patients undergoing skin graft harvest from the thigh for treatment of trauma, scars or tumours were included in this study. Patients with a history of hypersensitivity reaction to the topical formulation, those who were unwilling to participate in the study and those suffering from diseases that could interfere with wound healing, such as diabetes, renal failure, liver failure, malnourishment, cancer and hypoalbuminemia (serum albumin <4 g/dl), as well as elderly (age > 60 years) and pregnant patients, were excluded from the study (Fig. 1).

After the patients were administered general anaesthesia, the donor site was prepared with 10% povidone iodine solution. All skin grafts were harvested to a thickness of 0.4 mm from the antero-lateral thigh region with an electric Dermatome (Humeca).

Participants who met the eligibility criteria were randomized in to one of two groups by using a block randomization: *A. Tinctoria* cream (group A [*n* = 30]) and placebo cream (base cream without *A. tinctoria* extract, group B [*n* = 30]). The randomization sequence was generated by a statistician and maintained by an administrator who had no other involvement in the trial. One of the main researchers recruited patients on the basis of a randomization sequence. In the operating room, wounds were dressed and bandaged. The study group received topical *A. tinctoria* ointment daily, whereas the control group received daily topical application of placebo ointment (petrolatum gauze along with several sterile gauzes). The *A. tinctoria* ointment and placebo ointment were identical in weight, appearance, texture and colour. They were only recognizable by a code (A or B). The researchers and the subjects were blinded to the content of the ointment throughout the study and during the statistical analysis. After 2 days, according to the protocol, the donor site dressing was changed daily. At each visit, the percentage of healing at the donor site, as well as signs of infection, were recorded. The primary outcome measure was wound healing, which was assessed visually and photographically by the change in wound score according to the Bates-Jensen wound assessment tool at days 14 and 28 after the intervention. The outcome assessor was blinded to the participants’ allocation.

According to the sample size formula, we defined alpha (α) as the significance level (set at 0.05 consistent with 1–α or 95% in the context of confidence intervals) and beta (β) as the probability of making a type II error and standard deviation of the outcome variables. According to this formula and assumption of a 10% dropout rate, 30 patients in each group were considered. On the basis of the Bates-Jensen wound assessment between two groups in one week as a pilot study, μ_1_and μ_2_were also defined.

$$ \begin{array}{l}\alpha =0.05,\ \beta =0.001,{Z}^1- a/2=1.961150776,\ {Z}^1- B=3.090273048\\ {}{\mu}_1=8.6,\ {\mu}_2=28.6,\ {\sigma}_1=7.3,\ {\sigma}_2=7.4\\ {} n=\frac{{\left( z1-\frac{a}{2}+ z1-\beta \right)}^2\left(\sigma {1}^2+{\sigma}^22\right)}{{\left(\mu 1-\mu 2\right)}^2}\end{array} $$

### Preparation of Topical Formulation

Root of *A. tinctoria*, Vaseline, beeswax and sesame oil was used to formulate an *A. Tinctoria* (20%) ointment base. The final product was sterilized by gamma irradiation (25 kGy) to eliminate bacterial spores. The *A. tinctoria*-based ointment was provided by Daru Darman pharmaceutical lab (approved by the Iranian Health Authorities). A placebo ointment, consisting of the base formulation only without *A. tinctoria*, was also prepared.

### Toxicity Assessment

In a preliminary study, skin irritation due to this product was evaluated by the Driaiz Primary Skin Test [13, 14]. In this method, topical product was applied to the skin of animals (rabbits), and any signs of erythema and oedema were evaluated. No irritation (primary irritation index (PII) = 0) was observed in animals (data not shown).

### Wound Evaluation

Wound healing was assessed by the change in wound score according to the Bates-Jensen wound assessment tool. The Bates-Jensen wound assessment tool contains 13 items that assess wound size, depth, edges, undermining, necrotic tissue type, amount of necrosis, granulation and epithelialization tissue, exudate type and amount, surrounding skin colour, oedema and induration. These are rated on a modified Likert scale; the highest score indicates the unhealthiest wound. Each item was rated on a modified Likert scale and finally, the total score was obtained by adding together the 13-item scores. The Bates-Jensen wound assessment tool is available to use free with permission from the author at:

http://www.geronet.med.ucla.edu/centers/borun/modules/Pressure_ulcer_prevention/puBWAT.pdf)

In addition, signs of infection were assessed during the study.

### Data analysis

The data were analysed using SPSS version 18.0 software. Both the Student’s *t*- test and analysis of variance (ANOVA) were used to compare the wound score and wound surface area between the two groups and all two groups, respectively. The changes in the stage of healing during study were analysed by using repeated-measures ANOVA. Results were considered significant at *P* < 0.05. A chi-square test was used for descriptive variables.

## Results

Among the included patients, 30 patients (21 males and 9 females) in the *A. tinctoria* group and 30 patients (23 males and 7 females) in the control group completed the study (Fig. 1). There was no significant difference in the demographic data between the two groups (Table 1).

**Table 1:** Characteristics of the patients

Variable	Control group	*Alkanna tinctoria* group	*P*-value
Age, mean (SD), years	36.40 ± 11.48	37.13 ± 12.06	0.8
Sex			
Male, *n* (%)	22(73.3%)	21(70%)	0.7
Female, *n* (%)	8(26.6%)	9 (30%)	

To evaluate wound healing, the grade and surface area of the skin graft donor site were compared at days 14 and 28 after the intervention (Table 2). There was no significant difference in wound scores of both groups at baseline, but the decrease in wound scores was significantly higher in the *A. tinctoria* group as compared with the placebo group at days 14 and 28 after the dressing was applied. In addition, the wound surface area was significantly reduced in the *A. tinctoria* group as compared with the control group following 28 days of intervention. The mean widths of the scar were 7.37 ± 9.45 mm and 0.00 ± 0.00 mm in the case group, whereas these values were 53 ± 37.82 mm and 1.37 ± 3.43 mm in control group at 14 and 28 days, respectively (*P* < 0.05). No adverse effects were seen in either group treated with herbal and placebo ointments.

**Table 2:** Score comparison and surface area between the groups

Variable	Day^a^	Control group	*Alkanna tinctoria* group	*P*-value
Wound score^b^ mean ± (SD)	0	25.17 ± 7.42	25.07 ± 7.24	0.08
	14	20.63 ± 6.64	9.97 ± 1.30	0.001
	28	11.83 ± 2.77	9.03 ± 0.18	0.001
Patients with Wound score <10, *n* (%)	14	0	15 (50%)	0.001
	28	7(23.3%)	29(96.66%)	0.001

^a^Days after the intervention

^b^Score according to the Bates-Jensen wound assessment tool

## Discussion

The primary study outcome was the percentage change in wound score according to the Bates-Jensen wound assessment tool after 4 weeks of treatment with *A. tinctoria* extract ointment (20%) or standard daily dressing (dressing with base ointment) at the split-thickness skin graft donor site. Secondary outcomes included the percentage change in wound surface area, the proportion of patients who achieved complete healing and the adverse effects of dressing with *A. tinctoria* extract ointment. To the best of our knowledge, this is the first clinical study to evaluate the healing effect of *A. tinctoria* on the split-thickness skin graft donor site. Our results suggest that a dressing providing a moist environment gives beneficial effects [2, 15, 16]. Under optimal conditions, the skin graft donor site heals within 7 to 21 days [16]. A proper wound dressing may reduce the time to complete re-epithelialization and to prevent conversion of the donor site to a full-thickness wound [15, 17, 18]. The healing process occurs in three phases of inflammation, proliferation, and maturation [19, 20]. It is established that the inflammatory phase has the greatest impact on the healing process [21]. Alkannin, shikonin and their esters are the main ingredients in *A. tinctoria* that have anti-inflammatory, antimicrobial and antioxidant properties and promote wound healing [10, 12, 22]. Previous studies showed that alkannin/shikonin (A/S) had wound-healing effects on chronic ulcers, leprotic ulcers, burn injuries and anal fissures [10, 12]. As mentioned earlier, no studies have reported the wound-healing activity of A/Son split-thickness skin graft donor sites. The anti-inflammatory activity of A/S accelerates wound healing. In our study, decreases in wound score were significantly higher in the*A. tinctoria*group. More specifically, we found that the proportion of patients who achieved complete healing increased with use of the alkannin/shikonin (A/S)-based ointment. Other clinical studies showed that A/Shad healing effects in cases of chronic, severe or contaminated wounds. Furthermore, animal studies showed promotion of angiogenesis, collagen production and epithelialization in acute, non-contaminated wounds by the A/S-based ointment [23, 24]. Previously, it was indicated that the *Aloe vera* cream enhanced wound healing compared with dry gauze dressing; this may have been due to the moisture-retaining effect of *Aloe vera* on the wounds, but in the present study, it appears as though the pharmacologic properties of A/S were responsible for the observed effects [25]. These results indicate that wound-healing pharmaceutical preparations, such as A/S-based ointment, which modulates both inflammatory and proliferative phases of wound healing, maybe more efficient than standard dressing for donor site management after skin graft harvesting.

## Conclusion

A topical cream containing *A. tinctoria* exhibited the highest rates of complete healing, and wounds to which it was applied healed significantly faster than those bandaged with standard dressing.

### Limitations

In this study, pain scores during dressing changes were not evaluated. In addition, this short-term study did not enable us to evaluate scar appearance.

## Abbreviations

*A. tinctoria*: *Alkannatinctoria*
A/S: Alkannin and shikonin
STSG: Split-thicknesses skin grafts
